# Current Chemistry Investigators (CCI): Development and Evaluation of a Scientist in a Classroom Electrochemistry Workshop

**DOI:** 10.1021/acs.jchemed.3c00515

**Published:** 2023-09-12

**Authors:** John O’Donoghue, Natalia García Doménech, Fiona McArdle, Mary Connolly, Yvonne Lang, Niamh McGoldrick

**Affiliations:** † School of Chemistry, Trinity College Dublin, Dublin 2, Ireland; ‡ Department of Life Sciences, Atlantic Technological University, Sligo F91 YW50, Ireland

**Keywords:** Electrochemistry, Secondary Schools, Outreach, Researchers

## Abstract

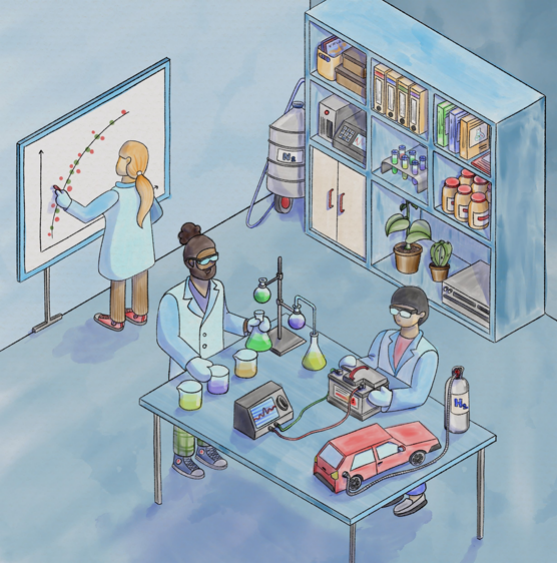

Over the course of the COVID-19 pandemic, school students suffered from a reduction in opportunities to connect with higher education institutions, meet scientific role models in person, discuss scientific career options, and carry out hands-on practical laboratory activities. Current Chemistry Investigators (CCI) is a successful electrochemistry-based STEM career intervention program, developed and evaluated through a co-creation process with teachers and students. The goals of CCI are 2-fold: first, to provide school students with career advice through tangible scientific role models and, second, to provide real-world context for the fundamentals of electrochemistry through hands-on activities. Herein, the development of a novel electro-analytical workshop from concept through to delivery with over a thousand students having taken part to date is reported. Students are tasked with solving why a battery malfunctioned through quantitative and qualitative analyses of an electrolyte using conductivity meters. Student feedback is also gathered anonymously through the use of a classroom response system (also known as “clickers”). Together with feedback from teachers, a robust evaluation is presented to measure the impact of providing tangible scientific role models and the usefulness of the workshop.

## Introduction

The 2019 Nobel Prize in Chemistry was awarded for the “*development of lithium-ion batteries*”, a breakthrough that has revolutionized society.^1^ The European Union and national Governments have committed to a carbon neutral climate, which will see a generational shift in how we convert, store, and use energy.^2,3^ Recent cost increases of fossil fuels have also raised questions about the prevalence of energy and transport poverty.^4^ Battery storage facilities are key to stabilizing the inconsistencies of renewable energy such as solar and wind, and there are plans and incentives in place to replace most fossil fuel cars with battery electric vehicles.^5−7^ Electrochemistry underpins the technology behind batteries, as well as analytical techniques such as glucose sensors, alcohol breath testers, heavy metal detection, and gel electrophoresis to name a few.^8,9^ As society becomes more reliant on electrochemistry-based technology, the Current Chemistry Investigators (CCI) program aims to increase interest in this area among school students as a positive action to address the likely increase in career demand for this area of chemistry in the near future.

Ireland has been at the forefront of electrochemical developments in recent history, from the Maynooth Battery (a 19th century commercially successful, large zinc–iron battery) to one of the first battery powered public trains in the world to modern cutting edge research.^10−13^ However, electrochemistry has been identified as one of the most difficult topics for chemistry learners, resulting in misconceptions, misunderstandings, and a perceived lack of relevance.^14−16^ In Ireland, it has also previously been found that electrochemistry is one of the least attempted topics on exams. Also, where students did attempt these questions, they answered very poorly.^17^ There have been numerous reports of educational interventions across the world covering a wide selection of electrochemistry concepts such as redox, potentiometry, and impedance, but they mainly centered around the structure and chemistry of batteries.^18−23^ To the best of our knowledge, there are no previous reports of an electro-analytical-based intervention program based on battery electrolytes for schools.

The CCI program uses a “*Scientist in a Classroom*” model, in which the expertise and enthusiasm of practicing scientists is used to aid learning, in what is sometimes referred to as the “third space”, i.e., a mix between formal and informal learning.^24,25^ Programs based on the “*Scientist in a Classroom*” model have frequently been reported to be successful by providing school students with tangible scientific role models and professional expertise through two-way communication.^24−27^ The social support provided by the role models can also “*exert a major influence on shaping careers*”.^28^ In addition, 85% of the population in Ireland believe that “*scientists have a professional responsibility to talk about research findings with the public*”.^29^ This model of engagement can also play a vital role in augmenting the socioeconomic backgrounds and the diversity of those interested in science as well as assist in the difficult school–university transition.^25,27,30^

The United Nations Education, Science and Cultural Organisation (UNESCO) Global Education Monitoring Report has called for the appropriate use of technology in education, which includes the use of smartphones in classrooms. It was found that, globally, almost one in four countries has a law or policy banning smartphone use in classrooms due to the disruption they cause.^31^ In addition to this, it was found from consultations with teachers that schools now use various systems to prevent smartphone use during class time.^32,33^ As a result, it is the view of the authors that QR codes, survey apps, and polling software are ineffective for evaluating school-based activities such as the one described herein. Instead, “classroom response systems” or “clickers” were identified for collecting pre- and post-workshop feedback surveys. Although previously reported extensively for higher education learning,^34−38^ the use of these systems for evaluation and for school learning is less common. Clickers provide student anonymity, interactivity, and reduced distractions compared to smartphone-based surveys due to the “single purpose nature” of the clicker devices.^38^ They also provide a key advantage over online pre- and post-evaluation surveys by anonymously tracking responses on a student-by-student basis for all questions through the assignment of an individual clicker number. They also do not require an Internet connection, which is a distinct advantage for running workshops in rural areas with poor coverage.

## Workshop Development

### Background

The target audience for the CCI workshop described herein is upper secondary (high) school students (16–18 years old). In Ireland, this consists of two distinct cohorts: (a) younger Transition Year (TY) students (16 years old) and (b) older fifth and sixth year Leaving Certificate (LC) students (17–18 years old). TY is a unique feature of the Irish secondary school system, representing an optional year of informal learning between the formal lower and formal upper secondary school curriculum. Depending on the school, TY students complete taster modules of subjects, undertake work placement, engage in community work, and complete school projects. TY students represent a broad spectrum of students who are, for the most part, yet to decide their specialization subjects (e.g., chemistry) for LC. TY has grown in popularity in recent years, with over 80% of students now opting to pursue it.^39^ LC students, on the other hand, are pursuing formal upper secondary education (2 years). In the case of this study, this cohort has chosen to study chemistry as one of their 4 or 5 specialized subjects.

The education system in Ireland therefore offers a unique opportunity to carry out an intervention and evaluation study with two distinct cohorts of students for comparison purposes–one who has already chosen to study chemistry and one who has yet to decide on whether to pursue further scientific study in formal education. Although nearly all students in Ireland (97%) receive an introduction to general science at lower secondary school, electrochemistry first appears on the curriculum at the LC level but still constitutes a relatively small area of study, with most of it appearing as optional content. As a result, in general, the younger TY cohort has no experience of electrochemistry prior to receiving a CCI workshop and very little experience of chemistry laboratory techniques. Analytical techniques on the other hand constitute a larger proportion of the LC chemistry curriculum in Ireland, as noted by teachers during our development consultations.^40^

**Figure 1:**
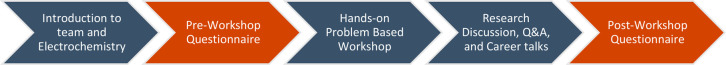
Summary outline of the CCI workshop structure.

During development of the CCI workshop, it was also noted that the word “ion” has entered a common lexicon in recent years through the widespread use of lithium-ion batteries. It was also noted that the word “electrolyte” has entered the common lexicon through the promotion of sports drinks. This provided a useful starting point for an introduction about the fundamentals of electrochemistry using real world examples (Figure 1). However, it was noted by teachers that there may be some misconceptions about ions and electrolytes among students. It was therefore decided to develop an “*electro-analytical*” workshop centered around the analysis of battery electrolytes. It is proposed here that this would assist student learning by bridging different areas of the curriculum. However, it should be noted here that electro-analytical chemistry does not formally appear on the Irish or UK curricula for schools.^40,41^ However, conductivity probes are listed on the International Baccalaureate (IB) program, alongside pH probes for determining acid strength and conductivity, and are also provided as an option for determining the end point of a titration in the College Board Advanced Placement (AP) Chemistry course.^42,43^

### Workshop Structure

Although the “*Scientist in a Classroom*” model traditionally involves a scientist traveling to a school to provide a workshop, it may also take place on a university campus to which the students travel. In the interest of flexibility and meeting the needs of individual schools, it was decided that both formats would be offered to schools where possible. The structure of the CCI workshop is outlined in Figure 1.

The scientists chosen to carry out the workshops are locally-based PhD students, henceforth termed PhD ambassadors to avoid any confusion with secondary students, although some faculty staff also attended a small number of workshops. Workshops are booked in advance with the teacher, after which a team of three PhD ambassadors are recruited from either of the two partner universities, depending on the location of the school. This results in an average ambassador:student ratio of about 1:8. During the delivery of the workshop, school students have numerous opportunities to talk with PhD ambassadors about their research on a one-to-one basis. At the end of the hands-on aspect of the workshop, a standalone discussion also takes place where the PhD ambassadors provide a 5 min overview of their research and answer questions about scientific careers, university life, career journeys to date, and career opportunities, as well as many other topics (Figure 1). This discussion is deliberately designed to be flexible to allow for unexpected questions and conversations with the students.

### Narrative

The use of a story or narrative can increase engagement in a topic through humanization and by providing context for abstract concepts.^44−47^ The story for the CCI workshop is based on a battery malfunction, providing the students with a topical, real-world problem to solve. It has been reported that lithium-ion batteries can fail due to variances in the concentration of electrolytes as well as due to the introduction of contaminants.^48^ The workshop presents the students with the problem of identifying why and how the battery malfunctioned, through a guided “electro-analytical” workshop consisting of two major parts: (1) a quantitative section and (2) a qualitative section. Three potential suspects are also presented to the students, who may be responsible for the malfunction of the fictional battery due to their responsibilities during the production of the battery.

### Quantitative

Students are accustomed to working in pairs on practical experiments at this level. Therefore, conductivity meters were identified as a desirable instrument for the workshop, since they are widely available as compact portable devices. This allows each team of students to have their own device for the duration of the workshop. It also allows transport of multiple devices easily and prevents any waiting by students to use the devices during the workshop.

In a solution of ions, the number of ions present is directly proportional to the conductivity of the solution.^49^ Therefore, the conductivity of a solution can be used to determine an unknown concentration by using a range of standards and a graph. The use of a calibration graph to determine an unknown quantity links with multiple parts of the curriculum in Ireland and elsewhere.^40,41^ Based on previous reports, standards of NaCl and LiCl were found to fit a linear regression with their associated conductivity and can be used to create a calibration graph to determine the concentration of an unknown sample as expected.^50^ Although the conductivity values differ slightly between LiCl and NaCl at similar concentrations, the resulting calibration graphs are very similar (See Figure S1). Therefore, it was determined that, from a cost and availability point of view, NaCl could easily be substituted for LiCl for an education-based workshop of this nature, without sacrificing student learning about ions and electrolytes.

**Figure 2:**
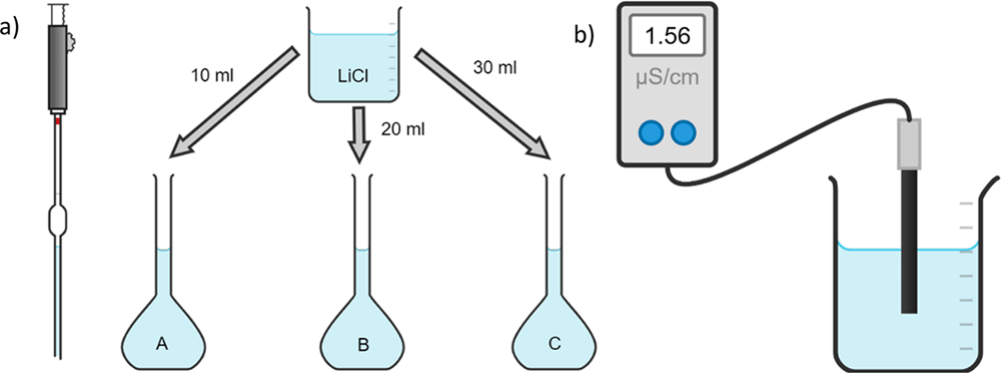
Schematic provided during the workshop of (a) the dilution from the 10.00 g/L stock solution and (b) measuring the conductivity of a sample.

The class of students are divided into teams of 2 or 3 students, with each team receiving a conductivity meter, a beaker, three 50 mL volumetric flasks, a 10 mL pipet, a pipet filler, and a wash bottle filled with water (tap water can be used in place of deionized water if required). Participants are then provided with a 10.00 g/L stock solution of LiCl (NaCl can be substituted for cost purposes), which they use to create 3 standards (Table 1). Due to the varying level of knowledge and experience of the students (especially among the younger TY cohort), it was decided that g/L should be used for all concentrations in the workshop to simplify the process; however, molar concentrations can be substituted here for more experienced groups as appropriate. Also, in the interest of time, 3 standards are employed during the workshop, but more can be added if time is not an issue (Table S1). Participants then use the provided conductivity meters to determine the conductivity of each standard and plot this against the concentration, obtaining a calibration graph with a line of best fit over the data (Figure S1). A schematic of the process, which is provided to the students, is presented in Figure 2.

**Table 1:** Standards Created by Diluting a 10.00 g/L Stock Solution of LiCl (NaCl Can Be Substituted for Cost Purposes) to Obtain the Calibration Graph

Sample	Volume from Stock Solution	Volume of Water	Final Volume	Concentration
A	10 mL	40 mL	50 mL	2.00 g/L
B	20 mL	30 mL	50 mL	4.00 g/L
C	30 mL	20 mL	50 mL	6.00 g/L

### Qualitative

Understanding and identifying the difference between quantitative and qualitative analysis is an important part of scientific learning and is required learning in many cases.^40−43^ During the development phase of the CCI workshop, it was determined that a potential scenario for the participants could be the identification of an unknown contaminant in a “battery sample” of electrolyte. A sample that resulted in an unexpected conductivity and/or concentration during a quantitative test would give students a logical reason to perform a qualitative test.

**Figure 3:**
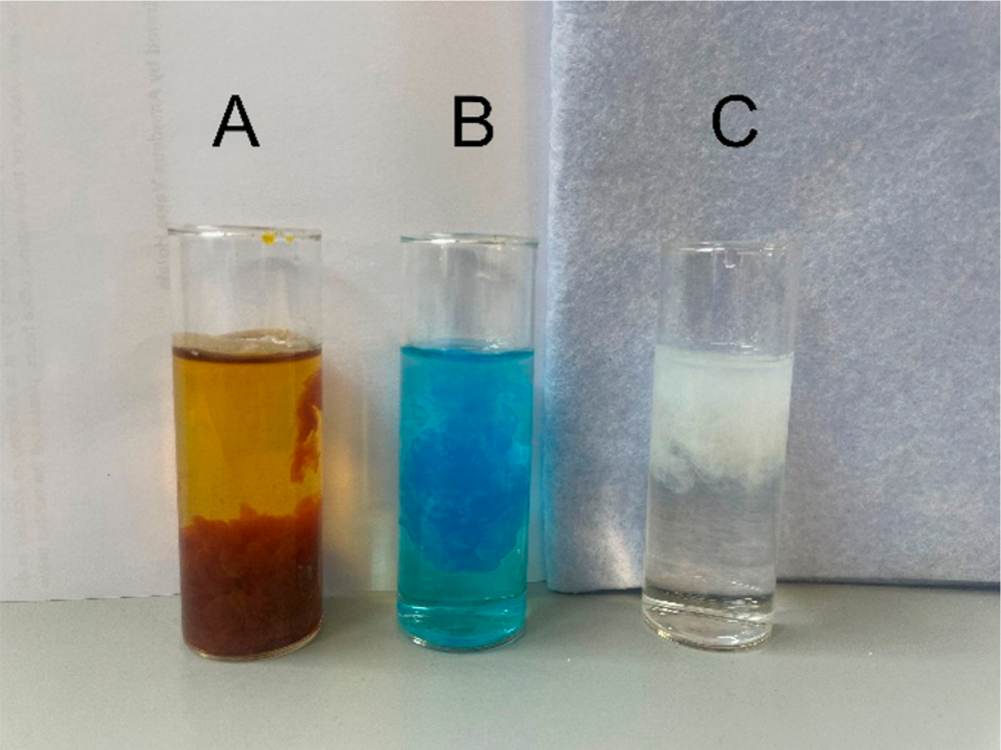
Precipitation of (A) iron(III), (B) copper(II) ,and (C) aluminum(III) after the addition of NaOH to a solution of LiCl/NaCl electrolyte spiked with the respective metal chlorides.

Aluminum, copper, and iron are some of the most common contaminants in lithium-ion battery electrolytes, since all three are already present within batteries.^48^ A qualitative test for aluminum, copper, and iron cations involves the use of NaOH to produce the equivalent metal hydroxides, which results in the formation of a precipitate. Iron(III) forms a yellowish-brownish precipitate; copper(II) forms a blue precipitate, and aluminum(III) forms a white precipitate (Figure 3). The equations of the precipitation reactions between the metal cations and NaOH are shown in eqs 1, 2, and 3.
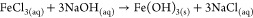
1
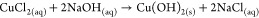
2
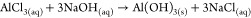
3

Spiking the sample with iron or copper chloride results in a colored solution before the addition of NaOH, making it overly obvious to the students that the unknown sample was spiked in comparison to the calibration standards. The use of AlCl_3_ on the other hand ensures that the sample remains colorless, making it difficult to differentiate it from the standards with the human eye; i.e., only the addition of the NaOH results in the formation of a precipitate (Figure 3).

**Figure 4:**
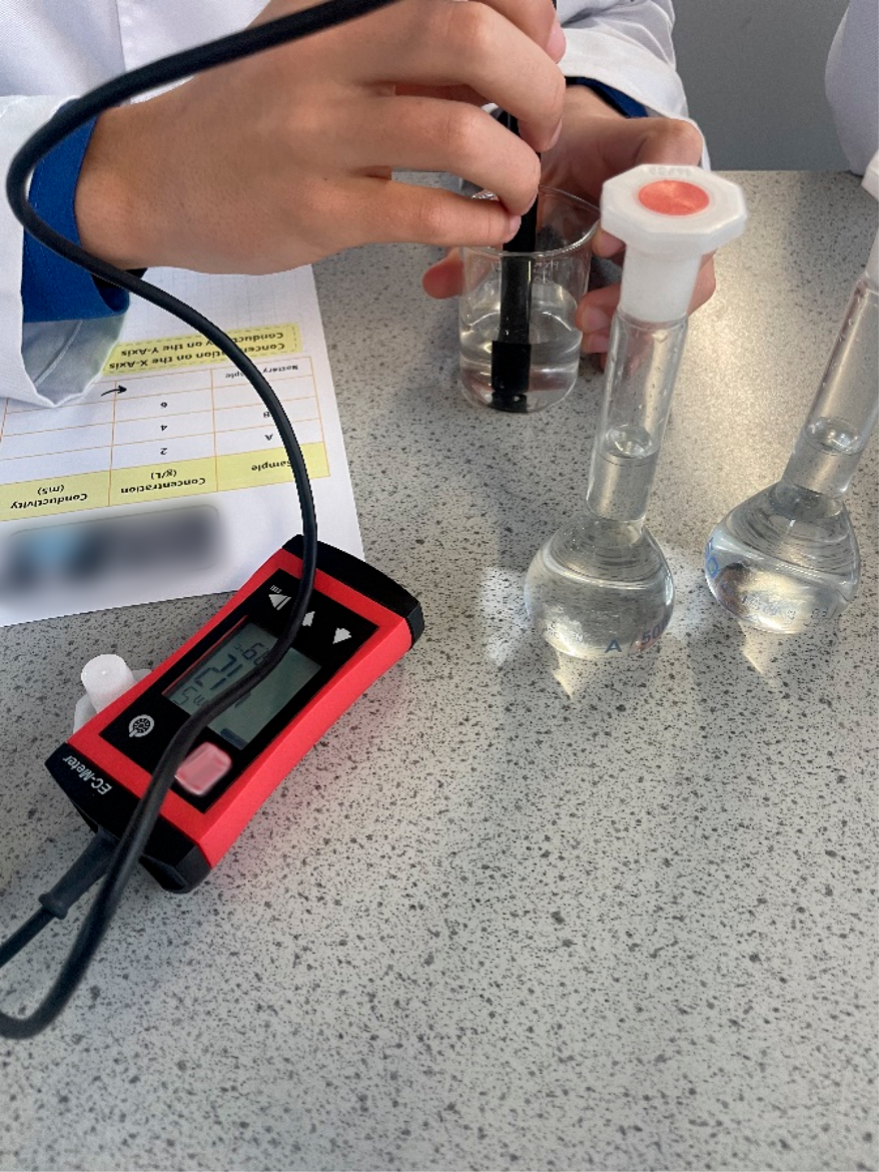
Student measuring the conductivity of a sample during a CCI workshop using a portable conductivity meter.

The effect of adding AlCl_3_ to a salt solution was tested by measuring the change in conductivity for different concentrations and examining the visibility of the precipitation reaction. After numerous tests during development to achieve the desired effect on a consistent basis, the stock solution of the “battery sample” is prepared before the workshop as 1.65 g of AlCl_3_·6H_2_O in 250 mL of a 1.00 g/L solution of LiCl/NaCl (a 10-fold dilution of the Stock Solution). This provides a conductivity reading of about 7.00 mS/cm, which falls within the range of the calibration graph from the quantitative section of the workshop. Upon completion of the calibration graph, 15 mL of the stock “battery sample” is provided to each team to measure its conductivity (Figure 4).

Using the calibration graph from the previous section, the participants can then work backward to find the concentration of the unknown “battery sample”. The teams of participants are told that the “battery sample” should have a concentration of 3.00 g/L but discover that it is in fact much higher than it should be at around 5.00 g/L. Following this discovery, a conversation with the students takes place about the possible reasons why this value is higher than expected, which should lead to the conclusion that a contaminant is present.

Three possible contaminants are then presented alongside the three associated suspects mentioned earlier in the narrative based on their contribution to the production of the battery. The students are also provided with information on how each metal contaminant interacts with NaOH. After a brief safety talk about the safe handling of harmful reagents, each team is then provided with 1 mL of 0.50 M NaOH in a plastic syringe (without a needle) to slowly add to their “battery sample” and instructed to observe any changes to the sample. Lower concentrations of NaOH were also found to work; however, the 0.50 M solution meant that only 1 mL needed to be provided, thereby reducing the risk of injury, and the Al(OH)_3_ precipitation occurs consistently with the desired visibility.

The addition of NaOH results in the formation of a white precipitate, which the students identify, using the information presented to them, is due to the presence of aluminum. This information is then used to work backward to identify the suspect who was working with aluminum during the battery production in the workshop narrative. Students are then encouraged to use the “classroom response system” (clickers) to “vote” for who they think is the person responsible, introducing further interactivity to the workshop since the results are presented to them on the screen for discussion. Further discussion about the use of the “classroom response system” (clickers) during the CCI workshop is provided in the next section.

## Feedback and Evaluation

### Background

Collecting reliable feedback was a core goal of the authors throughout the development of the CCI workshop. The use of paper-based surveys can provide good response rates but was ruled out due to the laborious nature of digitizing the data and the potential for transcription errors. Previous work by the authors with online digital surveys provided some success, but the response rates obtained from students after the workshop were less than desirable. Also, due to the desire for anonymity, there was no way of knowing if the same students completed the pre- and post-workshop surveys.^26^

The “classroom response system” or “clickers” employed here provide numerous distinct advantages over other methods, as discussed previously. Questions are embedded into the workshop presentation, allowing for data collection in conjunction with the workshop. The students are presented with multiple choice questions and are asked to enter the numbers of their desired answers on the clicker that they are assigned. The pre-workshop questionnaire was delivered between the initial workshop introduction and the background to the workshop, while the post-workshop questionnaire was delivered at the end of the session (Figure 1). In total, the use of the clickers only added around 10 min to the workshop time. The data were saved after each session as a spreadsheet file, allowing for immediate data processing. Unlike the interactive “voting” component employed during the workshop narrative, as discussed in the previous section, the responses to the survey questions were not presented to the students.

### Feedback Data

Pilot workshops were first run with 3 schools to gather suggestions from teachers and students about the workshop content, structure, and evaluation questions. The finalized workshop was then run during the school academic year, reaching a total of 1196 students in 48 schools. Data collected from the initial pilot workshops are not presented here since the workshop and questions underwent numerous changes based on the suggestions received from the teachers and students. Ethics approval for the evaluation was granted by the faculty of the STEM Research Ethics Committee in Trinity College Dublin. In accordance with previous reports and ethical guidelines, consent for running the survey with students was first obtained from the teacher or workshop facilitator through an online digital form before running the workshop.^51−53^ Where a teacher or facilitator did not provide consent, those students were omitted from this analysis.

Students in consenting schools were also asked directly for their consent, with 955 (80%) students consenting while 45 (4%) did not consent. The remaining 196 (16%) represent students who did not complete all of the questions during the workshop, resulting in their responses not being included here for consistency purposes. The gender identity provided by those who consented and completed all the questions was 51% female, 43% male, 3% nonbinary, 1% other, and 3% who prefer not to say. All percentages are rounded to the nearest whole number.

### Pre-workshop Student Questions

In addition to the consent and gender questions, two pre-workshop questions were asked of the students. The authors recognize that school students receive numerous external interventions at various stages of their formal education.^25,26,54,55^ The pre-workshop questions were therefore designed to establish a baseline for previous meetings with role models and the level of career encouragement received to date. This was deemed to be especially important due to the extensive disruption caused to these students by the COVID-19 pandemic.^56^

**Figure 5:**
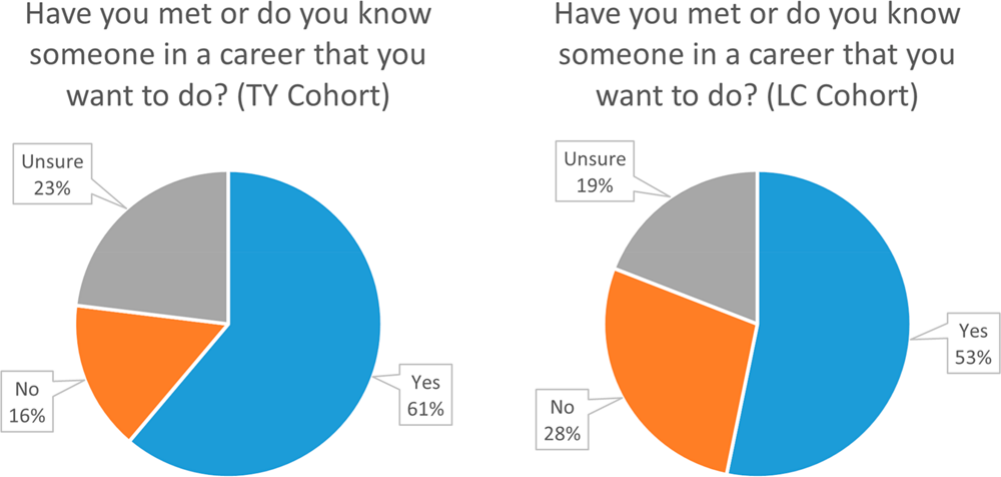
Responses to the pre-workshop questions “*Have you met or do you know someone in a career that you want to do?*” for the younger TY cohort (left) and the older LC cohort (right)

For the question “*Have you met, or do you know someone in a career that you want to do?*”, 57% of the total responded yes, while 43% stated that they had not previously met or known someone or were not sure. Interestingly, the age difference between the two student cohorts did not result in the older LC students being more likely to have previously met or know a role model (53%) compared to the younger TY group (61%), with the latter in fact being more likely to have met or know someone (Figure 5). In fact, a significantly larger number of the LC cohort reported that they had not met or knew a role model (28%) compared to the younger TY cohort (16%). It is likely that this represents a greater impact of the COVID 19 pandemic on the older LC cohort in relation to the opportunities and interventions available to them, since the LC cohort completed their own TY year during the time of most pandemic restrictions.^56^

The question “*How frequently has a family, friend, or school encouraged your career interests?*” resulted in only 10% of the total respondents answering never or rarely, with the remaining 90% answering sometimes, often, or always. The older LC student cohort was more likely to answer “always” to this question (28%) compared to the TY cohort (20%). Only small differences (1–3%) were found between the two cohorts for the other categories.

### Post-workshop Student Questions

After completion of the workshop (practical aspects and career discussion with the PhD ambassadors), three post-workshop questions were asked of the students (Figure 1). For the question “*Rate the workshop content in terms of how useful it was for your studies*”, it was found that 81% of the total students rated the workshop content as good or excellent. Only 4% of the students rated it as poor or very poor, while 15% of the students rated it as fair. Since the LC cohort formally study chemistry, it was unsurprising that they found the workshop more useful for their studies with 84% choosing good or excellent compared to 75% for the TY cohort. However, it is a surprise that such a large majority of TY students found it useful for their studies since they do not have a formal curriculum on which to base this question.

**Figure 6:**
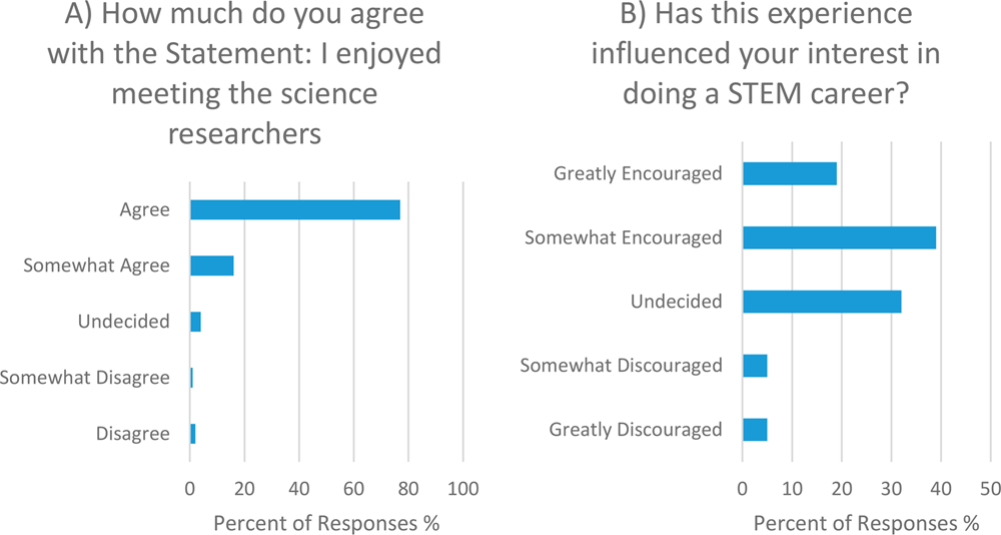
Responses to the post-workshop questions (A) “How much do you agree with the Statement: I enjoyed meeting the science researchers” and (B) “Has this experience influenced your interest in doing a STEM career?”.

The remaining two post-workshop questions (Figure 6) were designed to assess the impact of meeting the PhD ambassador role models and whether the workshop had an influence on the students in relation to their career aspirations. For the question “*How much do you agree with the Statement: I enjoyed meeting the science researchers*”, 93% of the total students stated that they somewhat agreed or agreed with the statement. No significant difference was found between the two cohorts for this question.

The last question asked the students, “*Has this experience influenced your interest in doing a STEM career?*”, where the definition of STEM was provided with the question as “Science, Technology, Engineering, and Maths” to avoid any confusion (Figure 6B). A total of 58% responded that the session had somewhat or greatly encouraged them to pursue a STEM career, with 32% being undecided. The remaining 10% of students felt somewhat or greatly discouraged by the CCI workshop to pursue a STEM career. Considering the stand-alone nature of the intervention described here, the overall positive impact on STEM career interest is impressive and noteworthy. It is also noteworthy that only a minority of students were discouraged by the experience, particularly since some interventions can have overall negative outcomes.^57^

A further breakdown of the responses to the last question (Figure 6B) revealed some key differences between the two student cohorts. First, the older LC cohort was significantly more likely to respond as somewhat or greatly encouraged by the CCI workshop experience to pursue a STEM career (69%) compared to the younger TY cohort (46%). It is worth noting here that, although the LC cohort is formally studying chemistry, they are also studying other subjects concurrently. Three of these are compulsory (English, Irish, and Maths) while a further three or four are chosen by the students. With 27% of the LC cohort responding to this question as undecided, the need for career-based interventions at all levels of school is clear regardless of whether the students have chosen to study STEM subjects or not.

In turn, the TY cohort was more likely to respond that they were somewhat or greatly discouraged by the experience (17%) compared to the LC cohort (5%), with the remainder being those who responded as undecided. Further analysis of the total “discouraged” TY cohort (17%) revealed that 11% responded “yes” to the pre-workshop question asking; “*Have you met, or do you know someone in a career that you want to do?*”. It is therefore very likely that the “discouraged” TY cohort made non-STEM related career decisions prior to the workshop, with the CCI workshop helping them finalize their decision in an informed manner. Interestingly though, since most TY students have yet to choose their subjects for formal study at the LC level, the vast majority had not ruled out a career in STEM with 46% stating that they were encouraged by the CCI workshop experience and 37% still undecided.

Finally, for the TY cohort, no significant difference in responses was found between the two majority genders identified by the students (male and female). However, for the LC cohort, interestingly, 77% of those who identified as female felt somewhat or greatly encouraged to pursue a STEM career due to the CCI workshop, compared to 61% for those who identified as male. It is the view of the authors that this may be due to the gender of the PhD researchers running the workshops, most of whom identify as female. However, confirmation of this link will be the subject of future work.

### Teacher Feedback

An online feedback form was also sent to teachers approximately 2 to 3 weeks after their students completed a CCI workshop, to allow time for the content and impact to be noticed. From a total of 48 schools, 27 teachers responded to the survey, resulting in a 56% response rate. Overall, the responses from teachers were very positive, with 93% stating that they were very likely to have their students participate in a similar session again in the future. The most common highlights mentioned by teachers included “lab practical skills”, “teamwork”, “real-life situations”, and “speaking with the PhD researchers”. They also rated the communication skills of the PhD researchers as mostly excellent (63%), and the remainder was divided between very good (33%) and fair (3%) with no negative ratings.

Teachers were also asked if they agreed or disagreed with three statements in relation to the workshop. For the first statement, “*This workshop increased your student’s awareness of the real-world applications of chemistry*”, 67% strongly agreed, 22% agreed, and only 11% disagreed. For the statement “*This workshop increased your student’s awareness of chemistry career options*”, 100% of the teachers either strongly agreed (74%) or agreed (26%). In response to the negative reverse statement “*There is little or no benefit for students to meet real-world science researchers*”, 75% responded that they strongly disagreed, 19% disagreed, and the remainder agreed (6%).

**Figure 7:**
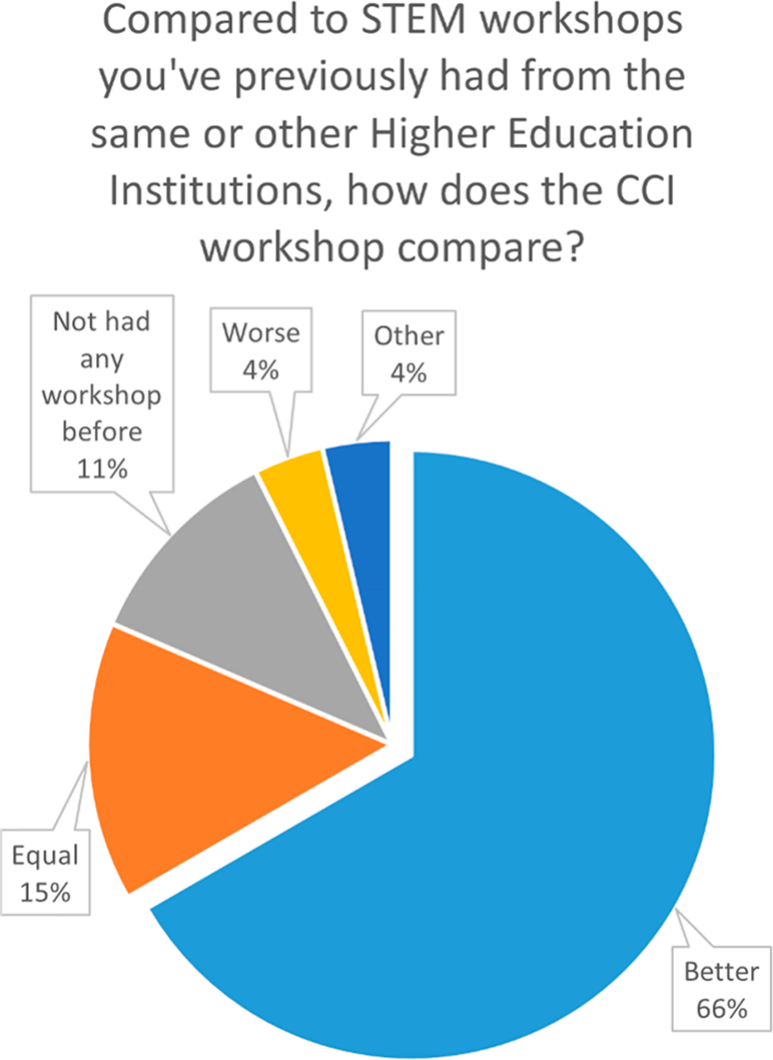
Teacher responses to the question “Compared to STEM workshops you’ve previously had from the same or other Higher Education Institutions, how does the CCI workshop compare?”.

As discussed previously, schools receive numerous external interventions annually from various organizations, particularly Higher Education institutions. Teachers were therefore asked to compare the CCI workshop to STEM workshops that they previously received from the same or other Higher Education Institutions (Figure 7). Encouragingly, 67% of the teachers stated that the CCI workshop was better, while 15% stated that it was equal; 11% did not have a workshop before, and the remainder thought it was worse or did not grade the workshop (7%).

Open ended text questions were also used such as “*Since completing the workshops, have you noticed any changes in your students in terms of their engagement?*”, which provided a range of responses. Summarizing the responses found that no difference or “N/A” was reported by 48% of teachers, but various examples of positive changes, albeit small in most cases, were reported by 52%. Teachers also provided some text-based feedback and suggestions about how to further improve the workshop, which will now be incorporated where possible for future delivery. Overall, teachers mostly praised the structure, content, and presenters in the open-ended text boxes with comments such as *Students have spoken openly about this workshop on how much they have enjoyed it and benefited from it. There has been more focus on practical work and the need to understand the theory*. Further comments are available in the Supporting Information.

## Conclusions

The CCI workshop is a successful STEM career and electrochemistry intervention program, with reliable digital feedback gathered from the outset through a successful co-creation process involving teachers and students. The goals set by the authors were to provide school students with career advice through tangible scientific role models and a real-world context for fundamentals of electrochemistry through hands-on activities. This study represents a thorough evaluation of the first goal of this program, namely, the career advice provided by tangible scientific role models. The impact evaluation of the workshop content and practical activities, particularly in terms of increased knowledge and understanding, is the subject of ongoing and future work. However, it is worth noting that the majority of students rated the workshop content as useful for their studies. The teacher feedback has also been very positive about the workshop content and the impact that it has already had on their students.

The use of the digital classroom response system, or “clickers”, has provided impressive levels of reliable data from the students. The digital nature of data collection has reduced the need for tediously transcribing from written forms and has reduced the possibility of errors. It also allowed for greater depth of analysis with larger numbers of respondents in a shorter period of time. However, the main disadvantage of the clicker system is the lack of open-ended text feedback. The number-based clicker devices only allow for specific numbered responses, which is the primary limitation of the system; however, the authors feel that the advantages of the clicker system in terms of the response rate, ease of use, reliability, digitization, and time savings outweigh this disadvantage.

The overwhelming positivity shown by the students toward meeting the PhD ambassadors demonstrates that the workshop has achieved its goal of providing the students with tangible scientific role models. The success of the workshop is also underpinned by the large number of students who felt encouraged to do a STEM career after the CCI workshop experience. Although a minority of students felt discouraged about STEM careers after the workshop, we felt that the anonymity provided by the clicker system encouraged students to provide honest responses to our questions. As a result, we feel that the negative responses actually provide evidence of robustness in our evaluation as well as strengthening the significance of the positive responses. It is the view of the authors that the CCI workshop still provided the “discouraged” students with the appropriate knowledge and experience to make an informed decision about their future, regardless of whether that involved a STEM career or not. The acronym STEM is explained to the students during the workshop as “*Science, Technology, Engineering and Maths*”. It was chosen to capture the broadest range of career possibilities influenced by the workshop content and discussions. However, for future feedback, it is now proposed to narrow this question to “Science” and/or “Chemistry” to compare the responses.

The target age group of students for this intervention has been greatly affected by the COVID 19 pandemic due to extensive disruptions to their formal and informal education. This includes reduced opportunities to meet role models through interventions such as those described herein. This is particularly evidenced by the LC cohort, who appear to have lost significantly more opportunities to meet role models and to have discussions with experts about STEM careers compared with their younger peers in the TY cohort. As a result, this report now represents the beginning of a longitudinal study to monitor the responses to the same questions over several academic years to observe the predicted drop-off in the COVID 19 influence. Further analysis of the data in terms of comparing school types, location of the school, and off-campus versus on-campus workshops is also the subject of ongoing and future work. This analysis will provide further detail about the impact of the CCI workshop experience so we can update the content, structure, and career discussions accordingly. Further evaluations are also planned with the PhD ambassadors to gather information about their experiences and how they have benefited from participation and delivery of the workshops.

## Supporting Information Available

Workshop development; feedback data; worksheet for students (PDF)
