# Supplementary material for: Current Chemistry Investigators (CCI): Development and Evaluation of a Scientist in a Classroom Electrochemistry Workshop
Source: J Chem Educ. 2023 Sep 12;100(10):4138–46. doi: 10.1021/acs.jchemed.3c00515 (PMC10571074; doi:10.1021/acs.jchemed.3c00515)
Supplement: Supplementary file 1 — ed3c00515_si_001.pdf [file ed3c00515_si_001.pdf]

Supporting Information

Current Chemistry Investigators (CCI): Development and Evaluation of a Scientist in a Classroom Electrochemistry Workshop

John O'Donoghue[1*], Natalia García Doménech,[1] Fiona McArdle[2], Mary Connolly[2], Yvonne Lang[2], Niamh McGoldrick[1].

[1] School of Chemistry, Trinity College Dublin, Dublin 2, Ireland

[2] Department of Life Sciences, Atlantic Technological University, Sligo, Ireland

AUTHOR INFORMATION

Corresponding Author

*E-mail: john.odonoghue@tcd.ie

WORKSHOP DEVELOPMENT

Comparison of salt standards:

Table S1. Concentration (M) and the corresponding conductivity (mS/cm) of NaCl and LiCl standards.

Sodium Chloride (NaCl) Standards			Lithium Chloride (LiCl) Standards		
**Sample**	**Concentration (M)**	**Conductivity (mS/cm)**	**Sample**	**Concentration (M)**	**Conductivity (mS/cm)**
**a**	0.02	2.21	**a**	0.02	1.87
**b**	0.04	4.23	**b**	0.04	3.45
**c**	0.06	6.09	**c**	0.06	5.07
**d**	0.08	8.01	**d**	0.08	6.61
**e**	0.10	9.70	**e**	0.10	8.14

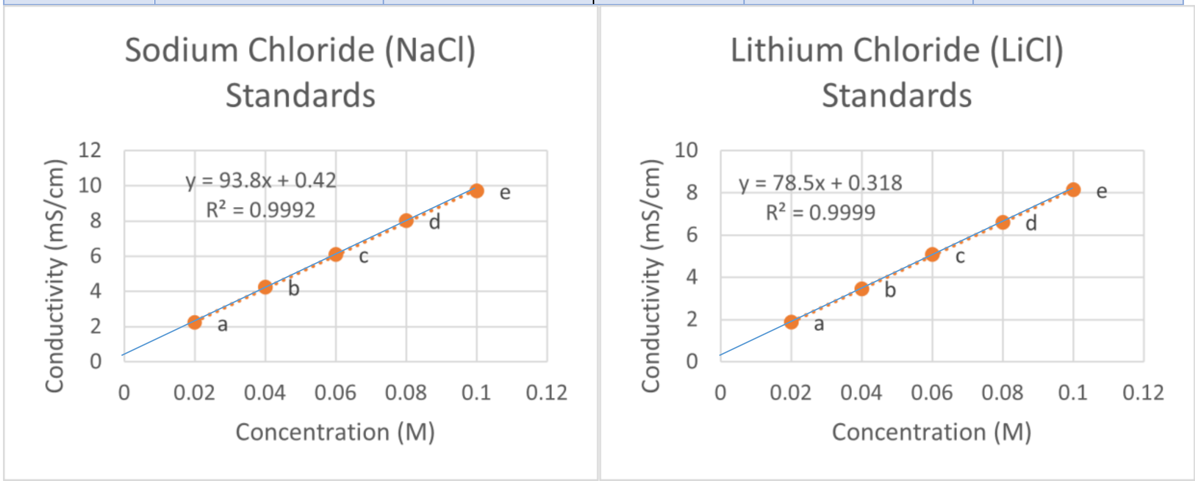

Figure S1. (left) a graph of conductivity vs concentration for standards of NaCl and (right), the same graph plotted using LiCl standards. Both are fitted through a linear regression.

Supporting Information

FEEDBACK DATA

Instructions Provided to the students:

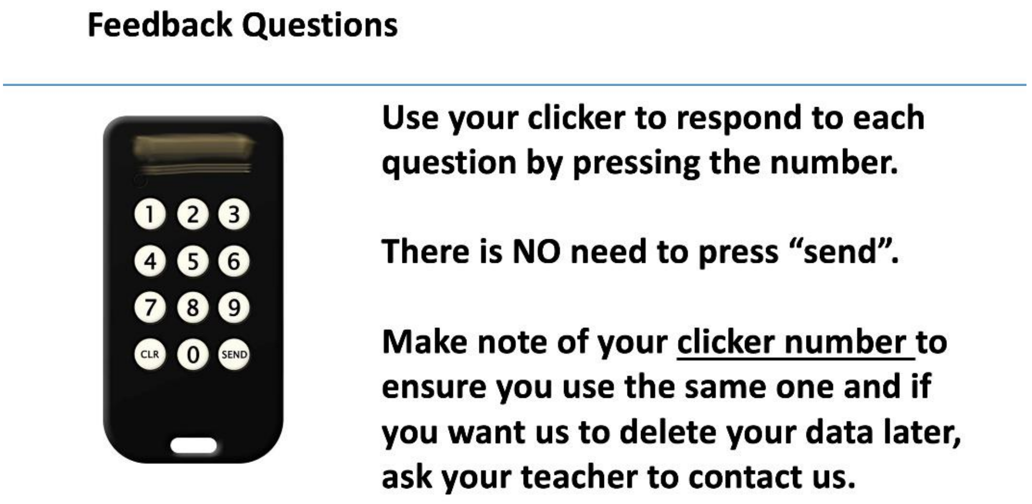

Figure S2. Image used during workshop to explain how to use the clickers.

Consent and Gender Questions:

The data presented here was collected from a total of 1196 students in 48 schools. Students in consenting schools were also asked directly for their consent with 955 (80%) students consenting, while 45 (4%) did not consent. The remaining 196 (16%) represent students who did not complete all the questions during the workshop resulting in their responses not being included here for consistency purposes.

If you agree to take part in the feedback study, please choose YES (1) on the clicker, acknowledging that:

I understand that the purpose of this study is to gather feedback about this workshop and am participating voluntarily. I am aware I can withdraw at any time, even after starting and I need to state my clicker number to do so. I understand that my anonymity will be ensured.

1) Yes
2) No

What Gender do you Identify as?

1. Female
2. Male
3. Non-Binary
4. Other
5. Prefer Not to Say

Pre-Workshop Student Questions:

Table S2. Pre-Workshop Questions Data					
**Have you met or do you know someone in a career that you want to do?**			**How frequently has a family, friend or school encouraged your career interests?**		
**Response Options**	**Number of Responses**	**Percent %**	**Response Options**	**Number of Responses**	**Percent %**

Supporting Information

**Yes**	547	57	**Never**	29	3%
**No**	205	22	**Rarely**	71	7%
**Not sure**	203	21	**Sometimes**	250	26%
			**Often**	365	38%
			**Always**	240	25%

Post-Workshop Student Questions:

**Table S3. Post-Workshop Questions Data for workshop content**		
**Rate the workshop content (1-5) in terms of how useful it was for your studies**		
**Response Options**	**Number of Responses**	**Percent %**
**1. Very Poor**	20	2%
**2. Poor**	15	2%
**3. Fair**	147	15%
**4. Good**	411	43%
**5. Excellent**	362	38%

**Table S4. Post-Workshop Questions Data for STEM careers**					
**How much do you agree with the Statement: I enjoyed meeting the science researchers (please rate 1-5)**			Has this experience influenced your interest in doing a STEM career (1-5)?		
**Response Options**	**Number of Responses**	**Percent %**	**Response Options**	**Number of Responses**	**Percent %**
**1. Disagree**	17	2%	**1. Greatly discouraged**	44	5%
**2. Somewhat disagree**	8	1%	**3. Somewhat discouraged**	44	5%
**4. Undecided**	43	4%	**2. Undecided**	306	32%
**5. Somewhat agree**	154	16%	**3. Somewhat encouraged**	375	39%
**6. Agree**	733	77%	**4. Greatly encouraged**	166	19%

Teacher Feedback:

If you agree to take part in the study, please click I Consent below and then begin the survey, acknowledging that: I understand the purpose of the study and am participating voluntarily. I am aware I can withdraw at any time, even after starting the survey. I understand that my anonymity will be ensured. I understand that I can withdraw permission to use my data within two weeks of completing each survey by emailing the researcher, in which case my response will be deleted. By clicking consent you also confirm your students participated in a Current Chemistry Investigators (CCI) workshop.

- I Consent
- I Do Not Consent

Table S5. Teacher responses to Likert questions	
**How likely are you to have your students participate in a similar session again in the future, if you had the option?**	
**Response Options**	**Number of Responses**
**Very Unlikely**	1
**Somewhat Unlikely**	0
**Neither Likely nor Unlikely**	0
**Somewhat Likely**	0
**Very Likely**	26
**This workshop increased your students awareness of the real-world applications of chemistry**	
**Response Options**	**Number of Responses**
**Strongly Agree**	18
**Agree**	6
**Neither Agree nor Disagree**	0
**Disagree**	0
**Strongly Disagree**	3
**There is little or no benefit for students to meet real-world science researchers**	
**Response Options**	**Number of Responses**
**Strongly Agree**	2
**Agree**	0
**Neither Agree nor Disagree**	0
**Disagree**	5
**Strongly Disagree**	20
**This workshop increased your student's awareness of chemistry career options**	
**Response Options**	**Number of Responses**
**Strongly Agree**	20
**Agree**	7
**Neither Agree nor Disagree**	0
**Disagree**	0
**Strongly Disagree**	0
**Compared to STEM workshops you've previously had from the same or other Higher Education Institutions, how does the CCI workshop compare?**	
**Response Options**	**Number of Responses**
**Better**	18
**Worse**	1
**Equal**	4
**Not had a workshop before**	3
**other**	1

**Table S6: Open text questions.**<br>**Are there any other areas that you feel this workshop has impacted?**
Appreciation of real world applications of chemistry
The simple graph was a great link to the maths syllabus . A lot of students can shy away from this in class at times .

Supporting Information

Teamwork, Communication
No
Identification and use of variables and introduction of errors. Drawing best fit graphs. Using graphs to solve problems.<br>Career options,
exposure to chemistry in real life terms. exposure to PHD students. really great experience for us all
Using graphs in 'real life' situations
My students enjoyed speaking with the PHD students who were there to deliver the workshop.<br>They found it inspiring to hear about the types of research they were doing and what it is like on a day to day basis and what it entails. It is so valuable for students to hear feedback from people who are actually doing this.
Good for overview of what chemistry is
They really enjoyed the discussion on third level and what studying Science is lile
College awareness
Curiosity and questioning - some more knowledge of research
Students could see where a career in science might take them through conversations with the PhD students.
We opted to have 5th and 6th years involved in the same workshop which was fantastic as the 6th years were able to help teach the 5th years. This increased their own confidence and was great for the 5th years to experience being taught by older students.
Relevance of science and technology in everyday life
Future career planning for students

**Since completing the workshops, have you noticed any changes in your students in terms of their engagement?**
Yes, they ask more questions about jobs in chemistry
If definitely gave them a confidence boost in their lab skills. The break from the ordinary class routine was also welcome. At times they can forget that people “do science” outside the walls of the school.
They enjoy learning about what is happening in the subject outside of the classroom such as new developments in green hydrogen and battery technology
Students have spoken openly about this workshop on how much they have enjoyed it and benefited from it. There has been more focus on practical work and the need to understand the theory.
There has been a slight increase in engagment by certain students but not the class as a whole
No.
Yes, they all wanted to research further aspects of the course, whether that was Chemistry of batteries, TCD Chemistry courses or career choices.
Not yet!
only had workshop today but time will tell
My students have expressed an interest in studying Chemistry at third-level. They have been asking more questions in class and they see the value of learning/using chemistry vocabulary and definitions to express ideas and concepts. They are more resilient and keep working when presented with challenging work.

Yes, with my 5th year group. They hadn't used any analytical equipment previously. When I introduced pipettes and pipettes fillers in class there was an air of anticipation in the group as they had seen them in your workshop and we anxious to get a go of them again.
More engaged
No
Yes. Students comfortable with using equipment. Some said it was useful for deciding to study LC Chem.
No. I have a good bunch who are very motivated
No, as it was only this week that we had the workshop.
Students asked questions about relating to science research as a career and employment possibilities for phd graduates. Workshop seems to have helped confidence in analytical skills.
N/A
The class are generally engaged and ask a lot of questions so I haven't noticed anything specific but I do feel it was a positive experience
N/A
Absolutely, their confidence has improved and they have asked more questions about chemistry and sciences outside the curriculum. My group were always quite engaged but the workshop resonated with them and they have been more inquisitive as a result. They are also more interested in where certain topics lead to in future study, such as organic chemistry. I think this is in part due to the discussion with the PhD researchers.
The students have been on work experience and on a overseas trip so I haven't engaged with them properly but the informal conversation after the workshop was very positive.
N/A
No
Students that enjoyed the workshop were more focused in Chemistry class
the LC students were already familiar in using the volumetric glassware in other contexts and were pleased to be able to use their skills in this workshop
Yes, particularly in the area of electrochemistry .It was great for them to see the real world application.

**What is the one thing that you have taken from this workshop that you will use with your students in the future?**
Multidisciplinary practical work
NA
It is a fantastic opportunity for our students to learn about the many exciting career opportunities that exist in chemistry!
An investigator approach
Basis operating of lab equipment - pipettes and measuring liquids accurately.
Quantitative analysis.
They really have no idea or experience of how open ended studying Science and Chemistry in particular is. Using simple problems to engage with topics. - Need to be prepared beforehand!
n/a
information about batteries and measuring conductivity of ions in solution
I continue to encourage students to explore their options and to consider studying chemistry or chemistry-related courses in university.

standard solutions and calibration curve.
Data analysis skills
I will incorporate this workshop into one of the assessments the students have to do on this course
Using calibration curves for a CSI activity
N/A
The problem solving aspect
Continued focus on graphing skills. Using a real life problem/mystery to engage students.
N/A
If time allowed use more problem solving techniques as in the workshop
N/A
The setup of the workshop was fantastic and just reinforced my goal of having equipment organised in sets for each group. The worksheet was also of high quality and very clear.
Promoting interactions with college students to allow conversations in the area of future careers
N/A
the broad range of electrochemistry experiments available for use
N/A
linking calibration curve of ion concentrations to colorimetric calibration curves
I will always ensure TY students carry out laboratory work and relate this work to research, careers in chemistry, real world issues ' sustainability etc

Worksheet for students:

This is the worksheet used for students during the workshop. It contains the details of the qualitative and quantitative components of the practical part of the workshop

Current Chemistry Investigators

The story:

- Prototype battery for smartphones caught on fire
- What happened?
- Concentration of electrolyte? **Part 1: quantitative analysis: Calibration graph**
- Is there a contaminant present? **Part 2: qualitative analysis: ion exchange**

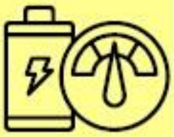

Definitions/keywords:

- **Ion:** atom or group of atoms which has either lost or gained electrons and has therefore a charge.
- **Electrolyte:** medium containing ions that is electrically conducting through the movement of ions, but not conducting electrons.
- **Conductivity:** measure of a material's ability to conduct an electric current.
- **Resistance:** measure of the opposition to current flow in an electrical circuit.

Part 1: Calibration graph

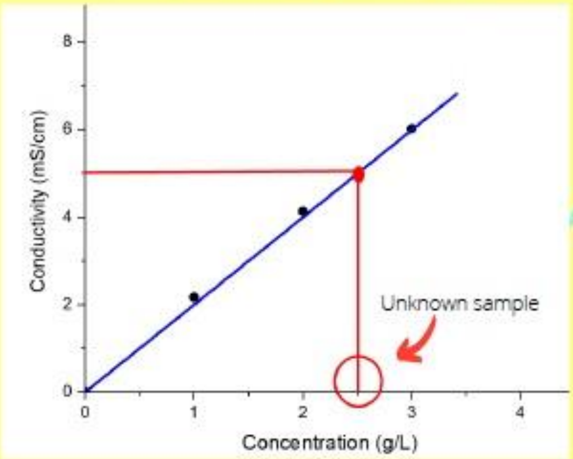

Part 2: Ion exchange

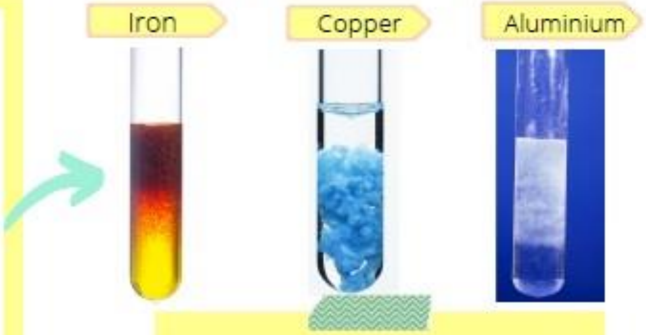

$FeCl_3 + 3NaOH \longrightarrow Fe(OH)_3 + 3NaCl$

$CuCl_2 + 2NaOH \longrightarrow Cu(OH)_2 + 2NaCl$

$AlCl_3 + 3NaOH \longrightarrow Al(OH)_3 + 3NaCl$

The suspects

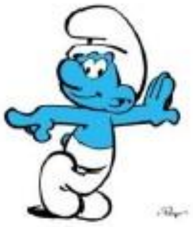

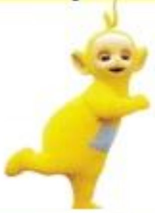

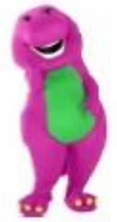

Dr. Blue was researching the battery casing which needs to be **strong**, they previously worked for a different battery company.

Dr. Yellow was researching ways to make the electrodes more **conductive**, she was spotted having an argument with the manager last week.

Mr. Purple was researching ways to make the battery much **lighter** for efficiency, he was fired from a previous job for unknown reasons.

Supporting Information

CCI
Current Chemistry Investigators

Trinity College Dublin
Coláiste na Tríonóide, Baile Átha Cliath
The University of Dublin

ATU
Ollscoil Teicneolaíochta an Atlantaigh
Atlantic Technological University

sfi
Fondúireacht Eolaíochta Éireann
Dá bhfuil romhainn
Science Foundation Ireland
For what's next

Sample	Concentration (g/L)	Conductivity (mS/cm)
A	2.00	
B	4.00	
C	6.00	
Battery sample		

1. Make up dilutions from the stock solution
2. Record the conductivity of the solutions
3. Plot a calibration graph of concentration vs conductivity
4. Measure conductivity of battery sample
5. Use the graph to find the concentration of the battery sample

Was the prototype battery tampered with?

Concentration on the X-Axis
Conductivity on the Y-Axis
